# Regulatory approval of new medical devices: cross sectional study

**DOI:** 10.1136/bmj.i2587

**Published:** 2016-05-21

**Authors:** Hani J Marcus, Christopher J Payne, Archie Hughes-Hallett, Adam P Marcus, Guang-Zhong Yang, Ara Darzi, Dipankar Nandi

**Affiliations:** 1 The Hamlyn Centre, Institute of Global Health Innovation, Imperial College, London W2 1NY, UK; 2 Department of Neurosurgery, Imperial College Healthcare NHS Trust, London, UK; 3 Faculty of Medicine, Imperial College, London, UK

## Abstract

**Objective** To investigate the regulatory approval of new medical devices.

**Design** Cross sectional study of new medical devices reported in the biomedical literature.

**Data sources** PubMed was searched between 1 January 2000 and 31 December 2004 to identify clinical studies of new medical devices. The search was carried out during this period to allow time for regulatory approval.

**Eligibility criteria for study selection** Articles were included if they reported a clinical study of a new medical device and there was no evidence of a previous clinical study in the literature. We defined a medical device according to the US Food and Drug Administration as an “instrument, apparatus, implement, machine, contrivance, implant, in vitro reagent, or other similar or related article.”

**Main outcome measures** Type of device, target specialty, and involvement of academia or of industry for each clinical study. The FDA medical databases were then searched for clearance or approval relevant to the device.

**Results** 5574 titles and abstracts were screened, 493 full text articles assessed for eligibility, and 218 clinical studies of new medical devices included. In all, 99/218 (45%) of the devices described in clinical studies ultimately received regulatory clearance or approval. These included 510(k) clearance for devices determined to be “substantially equivalent” to another legally marketed device (78/99; 79%), premarket approval for high risk devices (17/99; 17%), and others (4/99; 4%). Of these, 43 devices (43/99; 43%) were actually cleared or approved before a clinical study was published.

**Conclusions** We identified a multitude of new medical devices in clinical studies, almost half of which received regulatory clearance or approval. The 510(k) pathway was most commonly used, and clearance often preceded the first published clinical study.

## Introduction

The introduction of new medical devices is fundamental to the advancement of healthcare. Historically, such devices have been adopted with little scientific evidence to support their use.^1^ Although many devices have greatly improved clinical outcomes, not all are beneficial and some may be harmful. To this end most jurisdictions have developed regulatory bodies, such as the US Food and Drug Administration, that ensure the safety and effectiveness of new medical devices.^2^ These regulatory bodies must also act in an efficient and timely manner such that patients are not deprived from beneficial innovations.

The process by which new high risk medical devices find their way from bench to bedside is well established: the development of the device resulting in a first-in-human study; the evaluation of the device in clinical trials, culminating in a regulatory approval for use; and the adoption of the device.^3^ Although high risk devices warrant considerable scientific evidence for their safety and effectiveness before regulatory approval, the pathway for lower risk devices is less stringent, allowing for their more rapid approval.^4^ ^5^ ^6^

We investigated the use of these distinct regulatory approval pathways for new medical devices.

## Methods

We performed a cross sectional study of new medical devices reported in the literature to determine whether they received regulatory approval, and the relative contributions of academia and industry in this process. Before searching for evidence of regulatory approval, we identified clinical studies of devices, allowing us to capture those devices that failed to receive approval.

We defined a medical device according to the FDA definition as an “instrument, apparatus, implement, machine, contrivance, implant, in vitro reagent, or other similar or related article.” If there was no evidence of a previous clinical study of a device in the literature, we considered the device as new.

For each article reporting a clinical study of a new medical device, we defined academia and industry as being involved with the development of the device if a relation was described. If an entry could be found on the FDA medical device databases, we considered a device as having regulatory approval.

### Search strategy

The PubMed database was searched using the Boolean term: (device OR instrument OR apparatus OR implant OR “in vitro reagent” OR system) AND (“first in man” OR “first in human” OR “first experience” OR “first clinical” OR “early clinical” OR “early experience” OR “early human” OR “initial experience” OR “initial clinical” OR “initial human” OR “preliminary clinical” OR “preliminary experience” OR “preliminary human” OR “Phase 1” OR “Phase I”). We selected this search term owing to efficiency and being able to identify the most relevant studies. The search was carried out between 1 January 2000 and 31 December 2004 to allow time for regulatory approval, as previous studies have suggested a long lag between the development of a device and subsequent regulatory approval.^7^ ^8^

We included articles that reported a clinical study of a new medical device and excluded those that only reported a laboratory study of a device, because few such devices ultimately result in a clinical study.^9^ We also excluded articles if they reported on the novel use of an existing device, as we expected that most such devices would already have received regulatory approval.

Based on a pilot study, we estimated (between 1 January 2000 and 31 July 2000) that this search strategy would select sufficient articles to allow for meaningful analysis.

Two researchers initially screened titles and abstracts to identify relevant articles (HJM and CJP, checked by AHH and APM). We excluded articles if the title or abstract explicitly stated that the article was not original research, related to drug development, related to an existing medical device, or a laboratory study. Full articles were subsequently obtained and further assessed for eligibility. In each instance we reviewed the reference list and searched the PubMed database using the device name to ensure that we did not miss a related previous clinical study (that would result in their exclusion). Discrepancies were resolved by consensus.

### Medical devices

For each clinical study of a new medical device, we determined the type of device, the target specialty, and the involvement of academia and of industry (HJM and CJP, checked by AHH and APM). The types of device were based on the FDA definition, and the target specialties were drawn from the FDA databases. We considered academia and industry to be involved in the development of a device if a relevant author affiliation, financial support, or provision of technology was described in the author affiliations, main text, or acknowledgments of the article. Discrepancies were resolved by consensus.

### Regulatory approvals

For each new medical device, we searched the FDA databases for a relevant regulatory clearance or approval. The FDA recognises several types of regulatory pathway depending on the nature of the device. Premarket notification (510(k)) is the regulatory pathway if the device is “substantially equivalent” to a predicate device and does not necessarily require clinical data. Premarket approval is the regulatory pathway if the device is “not substantially equivalent,” and requires reasonable evidence of safety and effectiveness. Other regulatory pathways include humanitarian device exemption if the device is for use in patients with rare diseases or conditions. We searched the FDA 510(k), premarket approval, and humanitarian device exemption databases using the device name, applicant name, and relevant keywords (HJM and CJP, checked by AHH and APM). We also searched Google for devices that may have been discontinued, withdrawn, or recalled. Search results were not limited to a date range, allowing for the identification of regulatory clearance or approval before the first published clinical study. All the searches were performed in August 2015, allowing a minimum of 10 years from publication to regulatory clearance or approval. Discrepancies were resolved by consensus.

### Statistical analysis

To compare differences in regulatory clearance or approval between the following groups we used the χ^2^ test: devices developed by industry alone versus academia alone; devices developed by both industry and academia versus academia alone; and devices developed by both industry and academia versus industry alone. Firstly, we compared the proportion of devices receiving any regulatory clearance or approval (versus no clearance or approval). Secondly, we compared the proportion of devices receiving 510(k) clearance (versus any other approval). We considered differences to be statistically significant if P was less than 0.05. All statistical analyses were performed using SPSS 22.0 (IBM, NY, USA).

### Patient involvement

No patients were involved in setting the research question or the outcome measures, nor were they involved in developing plans for design or implementation of the study. No patients were asked to advise on interpretation or writing up of results. There are no plans to disseminate the results of the research to study participants or the relevant patient community.

## Results

### Search strategy

In all, 5574 titles and abstracts were screened, 493 full text articles assessed for eligibility, and 218 clinical studies of new medical devices included (fig 1). The corresponding authors originated from 28 countries, but most were located in the United States (70/218; 32%) and Germany (43/218; 20%).

**Figure f1:**
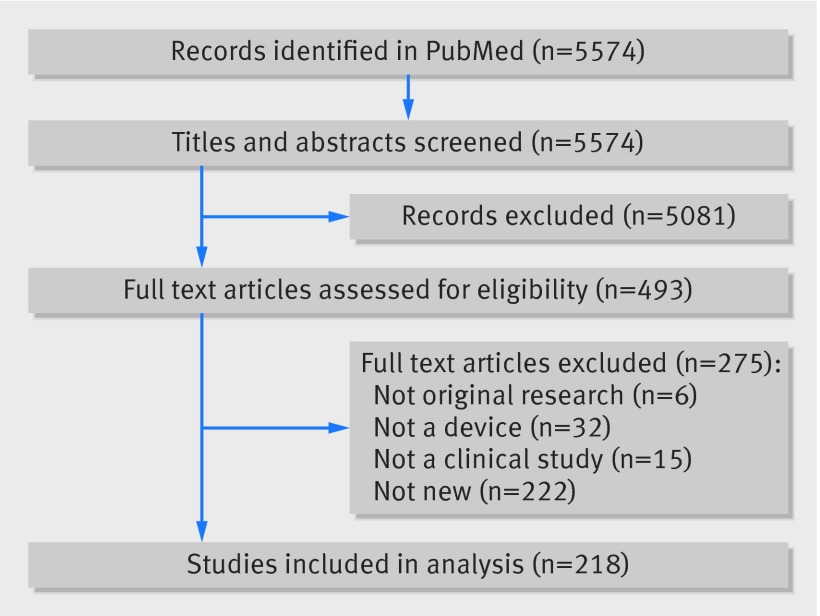
**Fig 1** Flowchart showing selection of clinical studies of new medical devices

### Medical devices

Most of the medical devices reported were instruments (86/218; 39%) or implants (79/218; 36%) (table 1). Devices were developed by industry alone (140/218; 64%), academia alone (46/218; 21%), or both (32/218; 15%).

**Table 1 tbl1:** Characteristics of new medical devices, and whether they ultimately received regulatory clearance or approval, or not

Characteristics	Total (n=218)	Clearance or approval (n=99)	No clearance or approval (n=119)
Type of device:			
Imaging	31	11	20
Implant	79	37	42
Instrument	86	47	39
Laboratory analysis	3	1	2
Monitor	10	3	7
Physiotherapy	7	0	7
Other	2	0	2
Target specialty:			
Anesthesiology	5	2	3
Cardiovascular	67	40	27
Clinical chemistry	2	0	2
Clinical toxicology	1	0	1
Dental	2	0	2
Ear, nose, and throat	12	3	9
Gastroenterology and urology	19	7	12
General and plastic surgery	22	11	11
General hospital	8	2	6
Haematology	2	1	1
Neurology	15	6	9
Obstetrics and gynaecology	11	6	5
Ophthalmology	11	5	6
Orthopaedics	22	10	12
Physical medicine	6	0	6
Radiology	13	6	7

### Regulatory approvals

Of the 218 devices described in clinical studies, 99 (45%) ultimately received regulatory clearance or approval (table 2). These included 510(k) clearance (78/99; 79%), premarket approval (17/99; 17%), and humanitarian device exemption (4/99; 4%).

**Table 2 tbl2:** Development of new medical devices, and whether they ultimately received regulatory clearance or approval, and regulatory pathway used

Developer	Total (n=218)	Clearance or approval (n=99)	510k (n=78)	Premarket approval (n=17)	Humanitarian device exemption (n=4)
Academia alone	46	5	5	0	0
Academia and industry	32	13	10	1	2
Industry alone	140	81	63	16	2

Regulatory clearance or approval was granted between April 1997 and September 2014. The median lag between publication of the clinical study and regulatory clearance or approval was 2 months (interquartile range −10.8 to 26.3 months). Of these, 43 devices (43/99; 43%) were actually cleared or approved before a clinical study was published; the median lag in these devices was −12.5 months (interquartile range −23.3 to −6.3 months).

Published clinical studies of devices that received regulatory clearance or approval were mostly case series’ comprising level 4 evidence (89/99; 90%).

### Statistical analysis

Devices were more likely to receive regulatory clearance or approval if developed by industry alone compared with academia alone (58% *v* 11%; P<0.001), or by both industry and academia compared with academia alone (41% *v* 11%; P=0.003). There was no significant difference in clearance or approval between devices developed by industry alone compared with both industry and academia (58% *v* 41%; P=0.114).

There was no significant difference in the proportion of 510(k) clearance and other approvals that were awarded to industry alone, industry and academia, or academia alone (P>0.1 in all cases).

## Discussion

We identified a multitude of new medical devices in clinical studies, almost half of which received regulatory approval. The 510(k) pathway was most commonly used, and devices often received regulatory clearance before the first published clinical study. The corollary is that many devices cleared for use in patients had no clinical data accessible in the literature to support their use. Published clinical studies were mostly case series’ comprising level 4 evidence. Without high quality clinical data available, informed shared decision making on the use of new medical devices is difficult if not impossible.

The 510(k) pathway is a fast track system that allows the regulatory approval of a device that is “substantially equivalent” to a predicate device. A device is considered substantially equivalent if it has the same intended use as the predicate device and it has the same technological characteristics, or, if it has different technological characteristics, information is provided that demonstrates that it is at least as safe and effective as the predicate device. Clinical studies are therefore not usually required.

The introduction of a device after it has been cleared through the 510(k) pathway is usually unstructured and variable.^2^ A device may be introduced in the form of a research study but, more often, may be published as a non-comparative trial without special institutional board review. Although many such devices are safe and effective, the dangers of this process are obvious and have been reported.^10^ ^11^ ^12^ ^13^ The Balliol Collaboration has proposed the IDEAL model for safe innovation to deal with this shortfall, the central tenet being that innovation and evaluation can and should proceed together in an ordered and logical manner.^2^ ^14^ ^15^ ^16^ ^17^ ^18^ Moreover, the FDA has recognised the need for reform and has announced a new vision for post-market surveillance of new devices.^19^

Industry was found to have a role in the development and regulatory approval of the majority of devices identified. For devices developed in academia, collaboration with industry was associated with greater regulatory approval. Interestingly, the proportion of 510(k), premarket approval, and other approvals that were awarded to industry and academia were comparable, suggesting that the greater regulatory approvals of devices developed by industry did not simply reflect a propensity for less disruptive and lower risk innovations. This finding supports efforts such as the Medical Device Innovation Consortium that facilitate collaboration among academia and industry to foster technology transfer.^20^ Collaboration between academia and industry may also contribute to improved surveillance of devices after regulatory approval has been received.

### Comparison with other studies

In keeping with the present study, several other groups have also found limited publically available evidence to support the regulatory clearance and approval of new devices. Zuckerman et al evaluated the types of scientific evidence used to support devices cleared using the 510(k) pathway.^5^ Of the 50 devices included, eight had data to support the claim that they were substantially equivalent to a predicate device, and only three had data on safety or effectiveness. Chang et al found that even devices approved using the premarket approval pathway, which require considerably more scientific evidence, often had no published clinical trials.^21^ When trials are published, comparators are often absent, and details may differ substantially from the data submitted to the FDA.^21^ ^22^

In a previous study we investigated the translation of new devices from the laboratory to first-in-human studies.^9^ In contrast with the present study we found that clinical rather than industry collaboration was the most important predictor of success; devices developed with clinical collaboration were over six times more likely to lead to a first-in-human study than those without. It is likely that this incongruity is the result of the varying role of clinical and industry collaboration through the device development pathway; early clinical studies may be more reliant on clinicians, and later regulatory approval more reliant on industry.

### Limitations of this study

We recognise several limitations to this study. We restricted our analysis to clinical studies of new medical devices reported in the biomedical literature. It is likely that the publication practices of academia and industry vary. We speculate that academia may be more motivated to publish early clinical studies.

Our analysis may also have favoured more novel devices, which clinicians might have thought warranted publication in the biomedical literature. The proportion of devices cleared through the 510(k) pathway was therefore likely to be an underestimate.

We determined whether a device had regulatory approval using only the FDA medical device databases. The proportion of medical devices receiving regulatory approval was therefore also undoubtedly an underestimate; in particular it is likely that licenses were granted from the European Union, which does not require any evidence of clinical value.^11^ The reason for selecting the FDA, rather than other licensing authorities, was because the FDA provides public databases and search engines that allowed for a systematic search strategy, the FDA acts as the central body for all medical devices receiving regulatory approval in the USA, and the USA represents the largest medical device market in the world. We hypothesise that most of the manufacturers of devices that received regulatory approval from another jurisdiction would have ultimately sought and obtained FDA approval within the timeframe of this study if they were successful.

We evaluated the contributions of academia and industry in the development of a device if a relation was described in the author affiliations, main text, or acknowledgments of the first published clinical study. We acknowledge that our cross sectional study design does not capture potential interactions between academia and industry during the early phase of a device’s development, such as the creation of spin-out companies or the licensing of intellectual property to industry. This study does not identify why industry was superior in obtaining regulatory approval compared with academia alone. One possible explanation is that the profit-seeking motive of industry hones its choice as to which devices are pursued.

### Conclusions

The optimal framework for the regulatory approval of medical innovations remains unclear. This study suggests that many new devices do receive regulatory approval but often lack clinical trial data supporting their safety and effectiveness.

The IDEAL model makes several proposals for the staged introduction of innovations in surgery (and other disciplines that offer complex interventions), including randomised controlled trials to assess safety and effectiveness. At present, few relevant randomised controlled trials are published, and fewer still meet current quality standards for optimal reporting. Changes in the regulatory approval of devices that would require trials for proof of safety and effectiveness might promote adherence to the IDEAL model.

**What is already known on this topic**

New medical devices have distinct regulatory approval pathways

**What this study adds**

Almost half of the new medical devices described in the literature ultimately receive regulatory clearance or approval
The 510(k) pathway (a fast track system allowing regulatory approval of a device that is “substantially equivalent” to a predicate device) is most commonly used, and clearance often precedes the first published clinical study

## Web extra

Extra material supplied by authors

Web appendix: supplementary information

## Notes

Ethical approval: Not required.

Data sharing: No additional data available.

Transparency: The lead authors (the manuscript’s guarantors) affirm that the manuscript is an honest, accurate, and transparent account of the study being reported; that no important aspects of the study have been omitted; and that any discrepancies from the study as planned have been explained.
