# Supplementary material for: Regulatory approval of new medical devices: cross sectional study
Source: BMJ. 2016 May 21;353:i2587. doi: 10.1136/bmj.i2587 (PMC4875244; doi:10.1136/bmj.i2587)
Supplement: Supplementary file 1 — Web appendix: supplementary information [file marh029502.ww1_default.pdf]

SUPPLEMENT

Table 1. Devices identified that received regulatory approval. * The Oxford 2011 Levels of Evidence

**Device**	**Article title**	**Journal**	**Year**	**Evidence**	**Level of evidence***
Talent abdominal stent graft system	Early experience with the Talent stent-graft system for endoluminal repair of abdominal aortic aneurysms.	Tex Heart Inst J	2000	Case series (n > 120)	Level 4
Cryogen cryosurgical system	Endometrial cryoablation with ultrasound visualization in women undergoing hysterectomy.	J Am Assoc Gynecol Laparosc	2000	Case series (n = 10)	Level 4
Debakey VAD	First clinical experience with the DeBakey VAD continuous-axial-flow pump for bridge to transplantation.	Circulation	2000	Case series (n = 2)	Level 4

Siemens magnetom 0.2T concerto	Interventional MRI-guided brain biopsies using inductively coupled surface coils.	Magn Reson Med	2000	Case series (n = 26)	Level 4
Plateletworks	Clinical evaluation of a new, point-of-care hemocytometer.	Crit Care Med	2000	Cross sectional study (n = 345)	Level 2
SMART nitinol stent system	Endovascular stenting for carotid artery stenosis: preliminary experience using the shape-memory- alloy-recoverable-technology (SMART) stent.	AJNR Am J Neuroradiol	2000	Case series (n = 4)	Level 4
HomMed sentry, Model 1 sentry	Emergence of electronic home monitoring in chronic heart failure: rationale, feasibility, and early results with the HomMed Sentry-Observer system.	Congest Heart Fail	2000	Non-randomised controlled cohort study (n = 53)	Level 3

Smith & Nephew HandPort system	Hand-assisted laparoscopic surgery (HALS) with the HandPort system: initial experience with 68 patients.	Ann Surg	2000	Case series (n = 68)	Level 4
EBI Omega 21 system	Biomechanical evaluation and preliminary clinical experience with an expansive pedicle screw design.	J Spinal Disord	2000	Case series (n = 14)	Level 4
MR elastography	High-resolution tensor MR elastography for breast tumour detection.	Phys Med Biol	2000	Case series	Level 4

5

ATTAIN access 6218 left-heart delivery system, model 6218	Initial results with left ventricular pacemaker lead implantation using a preformed "peel-away" guiding sheath and "side-wire" left ventricular pacing lead.	Pacing Clin Electrophysiol	2000	Case series (n = 13)	Level 4
Biologic-DT system (biologic-DT-1000 with DT-1000-TK)	Push-pull sorbent-based pheresis and hemodiabsorption in the treatment of hepatic failure: preliminary results of a clinical trial with the BioLogic-DTPF System.	Ther Apher	2000	Case series (n = 4)	Level 4
Lap discs	Hand assisted laparoscopic radical nephrectomy for renal carcinoma using a new abdominal wall sealing device.	J Urol	2000	Non randomised controlled cohort (n = 6)	Level 3

Gore helex™ septal occluder	Helex Septal Occluder for Closure of Atrial Septal Defects.	Curr Interv Cardiol Rep	2000	Case series (n = 28)	Level 4
Atlantis anterior cervical plate system	The management of one-level anterior cervical corpectomy with fusion using Atlantis hybrid plates: preliminary experience.	J Spinal Disord	2000	Case series (n = 8)	Level 4
P.D. access (percutaneous doppler) vascular access device	Gaining vascular access in pediatric patients: use of the P.D. access Doppler needle.	Catheter Cardiovasc Interv	2000	Case series (n = 39)	Level 4
Photon DR implantable cardioverter defibreillator (ICD)	Initial clinical experience with a dual chamber rate responsive implantable cardioverter defibrillator.	Pacing Clin Electrophysiol	2000	Case series (n = 57)	Level 4

Aescula LV model 1055K	Initial clinical experience with a new self-retaining left ventricular lead for permanent left ventricular pacing.	Pacing Clin Electrophysiol	2000	Case series (n = 13)	Level 4
Vasca LifeSite Hemodialysis Access System	Initial clinical results with the LifeSite Hemodialysis Access System.	Kidney Int	2000	Case series (n = 23)	Level 4
Omniport	Laparoscopic hand-assisted surgery for hepatic and pancreatic disease.	Surg Endosc	2000	Case series (n = 14)	Level 4
Ophthalmic medical laser system	Laser trabeculodissection with a photopolishing scanning excimer laser.	Ophthalmic Surg Lasers	2000	Case series (n = 8)	Level 4
SimpliCT	Potential of a new laser target system for percutaneous CT-guided nerve blocks: technical note.	Neuroradiology	2000	Case series (n = 8)	Level 4

Easytrak coronary venous steroid-eluding single-electrode	Transvenous left ventricular lead implantation with the EASYTRAK lead system: the European experience.	Am J Cardiol	2000	Case series (n = 186)	Level 4
Medtronic AVE solstice temporary occlusion balloon system	Balloon-assisted coil placement in wide-necked cerebral aneurysms: preliminary clinical experience.	Neurol Med Chir (Tokyo)	2000	Case series (n = 7)	Level 4
Leksell gamma knife target system, model 24001	First clinical experience with the automatic positioning system and Leksell gamma knife Model C. Technical note.	J Neurosurg	2000	Case series (n = 50)	Level 4

Cordis Palmaz Corinthian Transhepatic Biliary Stent and Delivery System	Initial experience using the Palmaz Corinthian stent for right ventricular outflow obstruction in infants and small children.	Catheter Cardiovasc Interv	2000	Case series (n = 4)	Level 4
Dysis	A novel optical imaging method for the early detection, quantitative grading, and mapping of cancerous and precancerous lesions of cervix.	IEEE Trans Biomed Eng	2001	Case series (n = 16)	Level 4
Sculptor robotic guidance arm (RGA)	The first clinical application of a "hands-on" robotic knee surgery system.	Comput Aided Surg	2001	Case series	Level 4

Cooltouch "v" Nd:YAG surgical laser	Facial rejuvenation with a nonablative 1320 nm Nd:YAG laser: a preliminary clinical and histologic evaluation.	Dermatol Surg	2001	Case series (n = 10)	Level 4
Excluder bifurcated endoprosthesis	Update on the bifurcated EXCLUDER endoprosthesis: phase I results.	J Vasc Surg	2001	Case series (n = 29)	Level 4
Contak TR pacemaker	[Experiences with a new transvenous electrode for left ventricular stimulation].	Herz	2001	Case series (n = 16)	Level 4
Symmetry	Sutureless mechanical anastomosis of a saphenous vein graft to a coronary artery with a new connector device.	Lancet	2001	Case study (n = 1)	Level 4

Gyrus plasmakinetic Superpulse System	Electrovaporization of the prostate with the Gyrus device.	J Endourol	2001	Case series (n = 42)	Level 4
Voice master prosthesis	First results of the VoiceMaster prosthesis in three centres in the Netherlands.	Clin Otolaryngol Allied Sci	2001	Case series (n = 85)	Level 4
Parietex composite (PCO) mesh	Laparoscopic repair of ventral and incisional hernias using a new composite mesh (Parietex): initial experience.	Surg Laparosc Endosc Percutan Tech	2001	Case series (n = 20)	Level 4
Polestar N-10	Novel, compact, intraoperative magnetic resonance imaging-guided system for conventional neurosurgical operating rooms.	Neurosurgery	2001	Case series (n = 20)	Level 4

Soundtec® direct system	Semi-implantable electromagnetic middle ear hearing device for moderate to severe sensorineural hearing loss.	Otolaryngol Clin North Am	2001	Case series (n = 5)	Level 4
Nit-occlud PDA	The duct-occlud device: design, clinical results, and future directions.	J Interv Cardiol	2001	Case series (n = 821)	Level 4
Ems swiss orthoclast	Cement removal with an endoscopically controlled ballistically driven chiselling system. A new device for cement removal and preliminary clinical results.	Arch Orthop Trauma Surg	2001	Case series (n = 28)	Level 4

Corlink Automated Anastomotic Device (AAD)	Early clinical experience with a new sutureless anastomotic device for proximal anastomosis of the saphenous vein to the aorta.	J Thorac Cardiovasc Surg	2001	Case series (n = 17)	Level 4
Visian ICL (implantable collamer lens)	Collamer intraocular lens: clinical results from the US FDA core study.	J Cataract Refract Surg	2001	Case series (n = 686)	Level 4
Ligasure Vessel Sealing System	Initial results with an electrothermal bipolar vessel sealer.	Surg Endosc	2001	Case series (n = 98)	Level 4

Siremobil ISO-C 3D	[3-D imaging with a mobile surgical image enhancement equipment (ISO-C-3D). Initial examples of fracture diagnosis of peripheral joints in comparison with spiral CT and conventional radiography].	Unfallchirurg	2001	Cross sectional study	Level 2
Safe-steer guide wire system	Initial experience and safety in the treatment of chronic total occlusions with fiberoptic guidance technology: optical coherent reflectometry.	Catheter Cardiovasc Interv	2001	Case series (n = 6)	Level 4
Extracorporeal shock wave lithotripter	The first clinical results of "wide-focus and low-pressure" ESWL.	Ultrasound Med Biol	2002	Case series (n = 297)	Level 4

GE discovery LS system	Initial clinical experience using a new integrated in-line PET/CT system.	Br J Radiol	2002	Case series	Level 4
Shelhigh no-react tissue repair patch/uropatch.	The YAMA UroPatch sling for treatment of female stress urinary incontinence: a pilot study.	J Laparoendosc Adv Surg Tech A	2002	Case series (n = 22)	Level 4
Medtronic model 7272 InSync ICD	Initial experience with an implantable cardioverter-defibrillator incorporating cardiac resynchronization therapy.	J Am Coll Cardiol	2002	Case series (n = 84)	Level 4
Mammosite radiation therapy system (RTS) tray, mammosite HDR afterloader accessories tray	Dosimetric characteristics of the MammoSite RTS, a new breast brachytherapy applicator.	Int J Radiat Oncol Biol Phys	2002	Case control study (n = 12)	Level 4

Coalescent U-clip delivery and disposal device	Early experience of coronary artery bypass grafting with a new self-closing clip device.	J Thorac Cardiovasc Surg	2002	Case series (n = 14)	Level 4
X-sept transseptal sheath and transition catheter, model mv-03-09-90, mv-03-10-90, mv-03-11-90, mv-03-09-120, mv-03-10-1	Percutaneous left atrial appendage transcatheter occlusion to prevent stroke in high-risk patients with atrial fibrillation: early clinical experience.	Circulation	2002	Case series (n = 15)	Level 4
X-sizer catheter system	Early experience with a helical coronary thrombectomy device in patients with acute coronary thrombosis.	Am J Hematol	2002	Case series (n = 35)	Level 4
St. Jude medical regent mechanical heart valve (aortic)	Experimental evaluation and early clinical results of a new low-profile bileaflet aortic valve.	Artif Organs	2002	Case series (n = 30)	Level 4

Nomos corvus 5.0m	Clinical implementation of intensity-modulated arc therapy.	Int J Radiat Oncol Biol Phys	2002	Case series (n = 50)	Level 4
Boston keratoprosthesis or Boston KPRO	Seoul-type keratoprosthesis: preliminary results of the first 7 human cases.	Arch Ophthalmol	2002	Case series (n = 7)	Level 4
Valleylab ligasure precise instrument vessel sealing system-model # ls1200 & sligaure generator	Use of a bipolar vessel-sealing device for parenchymal transection during liver surgery.	J Gastrointest Surg	2002	Case series (n = 27)	Level 4
Intrastent doublestrut stent	Initial experience with intratherapeutics Intrastent Doublestrut LD stents in patients with congenital heart defects.	Catheter Cardiovasc Interv	2002	Case series (n = 22)	Level 4

Niti-s stent & introducer, model eoxxxx	Polyurethane-covered self-expandable nitinol stent for malignant biliary obstruction: preliminary results.	Cardiovasc Intervent Radiol	2002	Case series (n = 10)	Level 4
Artifical cervical disc	Preliminary clinical experience with the Bryan Cervical Disc Prosthesis.	Neurosurgery	2002	Case series (n = 60)	Level 4
The auto suture MIBB system	Stereotactic breast biopsy with an 8-gauge, directional, vacuum-assisted probe: initial experience.	Eur Radiol	2002	Case series (n = 138)	Level 4
Biorigid nail femur (BNF)	["Biorigid" interlocking after unreamed intramedullary nailing of tibial shaft fractures].	Unfallchirurg	2002	Case series (n = 76)	Level 4

Macropore hydrosorb spine system	Resorbable polymer implants in unilateral transforaminal lumbar interbody fusion.	J Neurosurg	2002	Case series (n = 60)	Level 4
Boston scientific filterwire ex embolic	Initial clinical experience with distal protection using the FilterWire in patients undergoing coronary artery and saphenous vein graft percutaneous intervention.	Catheter Cardiovasc Interv	2002	Case series (n = 35)	Level 4
Storz millennium microsurgicl system high speed vitrectomy system	Initial experience using the transconjunctival sutureless vitrectomy system for vitreoretinal surgery.	Ophthalmology	2002	Case series (n = 33)	Level 4

Eg-3630ur, ultrasund video gastroscope	Initial experience with an electronic radial array echoendoscope: randomized comparison with a mechanical sector scanning echoendoscope in humans.	Gastrointest Endosc	2002	Cross sectional study (n = 14)	Level 2
Bodyfix	A novel vacuum device for extremity immobilisation during digital angiography: preliminary clinical experiences.	Eur Radiol	2002	Non randomised controlled cohort (n = 100)	Level 3
Setpoint endovascular temperature management system	Initial experience with a novel heat-exchanging catheter in neurosurgical patients.	Anesth Analg	2002	Case series (n = 8)	Level 4
HTS coil	Superconducting RF coils for clinical MR imaging at low field.	Acad Radiol	2003	Cross sectional study	Level 2

Safe-cross deflecting catheter, model c114nd1	Initial experience and safety in the treatment of chronic total coronary occlusions with a new optical coherent reflectometry-guided radiofrequency ablation guidewire.	Am J Cardiol	2003	Case series (n = 13)	Level 4
Tissuelink monopolar floating ball	Early experience employing a linear hepatic parenchyma coagulation device.	J Hepatobiliary Pancreat Surg	2003	Case series (n = 7)	Level 4
Endoscopic plication system	Endoscopic full-thickness plication: the device, technique, pre-clinical and early clinical experience.	Gastrointest Endosc Clin N Am	2003	Case series (n = 6)	Level 4

Surgical sealant	Feasibility study of NeoMend, a percutaneous arterial closure device that uses a nonthrombogenic bioadhesive.	AJR Am J Roentgenol	2003	Case series (n = 26)	Level 4
Daum-lectric MRI drilling machine	Magnetic resonance-guided transcortical biopsy of bone marrow lesions using a magnetic resonance imaging-compatible piezoelectric power drill: preliminary experience.	Invest Radiol	2003	Case series (n = 17)	Level 4
Spy intra-operative imaging system: sp2000	Preliminary experience with a novel intraoperative fluorescence imaging technique to evaluate the patency of bypass grafts in total arterial revascularization.	Ann Thorac Surg	2003	Case series (n = 84)	Level 4

CV232 sre pre-rolled acrylic intraocular lens	Deep sclerectomy with a nonabsorbable implant (T-Flux): preliminary results.	Can J Ophthalmol	2003	Case control study (n = 25)	Level 4
Reform peripheral catheter system, model 02200; reform peripheral cathetercatheter, model 02406	Initial experience with a new 8 French-compatible directional atherectomy catheter: immediate and mid-term results.	Catheter Cardiovasc Interv	2003	Case series (n = 77)	Level 4
Surgifrost 10 cm cryosurgical device plus frostbyte clamp and cryosurgical console	Intraoperative left atrial ablation (for atrial fibrillation) using a new argon cryocatheter: early clinical experience.	Ann Thorac Surg	2003	Case series (n = 28)	Level 4
Attain 6218a-am amplatz guide catheter for left-heart delivery	New catheter design for cannulation of the anomalous right coronary artery arising from the left sinus of valsalva.	Catheter Cardiovasc Interv	2003	Case series (n = 5)	Level 4

Rossmax automatic blood pressure monitor, model cardiocare 1000i	Validation of the ROSSMAX blood pressure measuring monitor according to the European Society of Hypertension International Protocol for Validation of Blood Pressure Measuring Devices in Adults.	Blood Press Monit	2003	Cross sectional study (n = 33)	Level 2
Tonoport V	Validation of the TONOPORT V ambulatory blood pressure monitor according to the European Society of Hypertension International Protocol for Validation of Blood Pressure Measuring Devices in Adults.	Blood Press Monit	2003	Cross sectional study (n = 33)	Level 2

Neuroform™ microdelivery stent system	Preliminary experience using the Neuroform stent for the treatment of cerebral aneurysms.	Neurosurgery	2004	Case series (n = 18)	Level 4
Trellis infusion system (10cm infusion length); trellis infusion system (20cm infusion length)	Clinical and economic evaluation of the trellis thrombectomy device for arterial occlusions: preliminary analysis.	J Vasc Surg	2004	Case series (n = 26)	Level 4
ATS 3f aortic bioprosthesis	Early clinical experience with a new tubular equine pericardial stentless aortic valve.	Heart Surg Forum	2004	Case series (n = 24)	Level 4
Portaclamp	Early experience with a new aortic clamping system designed for port access cardiac surgery: the PortaClamp.	Heart Surg Forum	2004	Case series (n = 20)	Level 4

Silverhawk peripheral plaque excision system, models 02550,04800, 05200, 02406, 04706, 04300	Early experience with a novel plaque excision system for the treatment of complex coronary lesions.	Catheter Cardiovasc Interv	2004	Case series (n = 10)	Level 4
Corlink AAD (3.5 to 6.0 m m outer diameter vessels),model 200-064, corlink aad (2.0 to 4.0 mm outer diameter vessels), m	Initial experience of an automated anastomotic distal device in off-pump CABG.	Heart Surg Forum	2004	Case series (n = 14)	Level 4
Abiocor® Implantable Replacement Heart	Initial experience with the AbioCor implantable replacement heart system.	J Thorac Cardiovasc Surg	2004	Case series (n = 7)	Level 4
Medamicus flowguard peelable introducer	Preliminary evaluation of a valved introducer sheath for the insertion of tunneled hemodialysis catheters.	Semin Dial	2004	Case series (n = 15)	Level 4

Levitronix centrimag extracorporeal blood pumping system, model l-100	The CentriMag: a new optimized centrifugal blood pump with levitating impeller.	Heart Surg Forum	2004	Case series (n = 11)	Level 4
Outback catheter	The outback catheter: a new device for true lumen re-entry after dissection during recanalization of arterial occlusions.	Cardiovasc Intervent Radiol	2004	Case series (n = 10)	Level 4
Gambro prismaflex and gambro prismaflex m60 & m100 sets	First clinical trial for a new CRRT machine: the Prismaflex.	Int J Artif Organs	2004	Case series (n = 13)	Level 4
Impella recover LP 2.5 percutaneous cardiac support system	Initial experience with miniature axial flow ventricular assist devices for postcardiotomy heart failure.	Ann Thorac Surg	2004	Case series (n = 6)	Level 4

Biopsy handy, MRI biopsy handy	A new safe and stable spiral wire needle for thoracoscopic resection of lung nodules.	Chest	2004	Case series (n = 13)	Level 4
Contegra® Pulmonary Valved Conduit, Models 200 (unsupported) and 200S (supported)	Contegra pulmonary valved conduits cause no relevant hemolysis.	J Card Surg	2004	Case series (n = 60)	Level 4
Microcuff pediatric endotracheal tube	Tracheal sealing characteristics of pediatric cuffed tracheal tubes.	Paediatr Anaesth	2004	Randomised trial (n = 80)	Level 2
Cardiovention corx system, model FG 0001	A new cardiopulmonary bypass circuit with reduced foreign surface (CorX): initial clinical experience and implications for anaesthesia management.	Eur J Anaesthesiol	2004	Case series (n = 10)	Level 4

ACMI vista CTR bipolar loop electrode	First clinical experience with new transurethral bipolar prostate electrosurgery resection system: controlled tissue ablation (coblation technology).	J Endourol	2004	Case series (n = 36)	Level 4
MO.MA ultra proximal cerebral protection device, model mus0130069x6	First clinical experiences with an endovascular clamping system for neuroprotection during carotid stenting.	Eur J Vasc Endovasc Surg	2004	Case series (n = 42)	Level 4
1.5T 32-channel head coil and 3T 32-channel head coil	New partially parallel acquisition technique in cerebral imaging: preliminary findings.	Eur Radiol	2004	Case series (n = 6)	Level 4

Box 1. FDA processes.

510(k) is a premarketing submission to demonstrate that a device is as safe and effective, that is “substantially equivalent”, to a legally market device.

Premarket Approval (PMA) contains sufficient valid scientific evidence to provide reasonable assurance that the device is safe and effective for its intended use or uses.

Humanitarian Use Device (HUD) is similar to PMA, but is exempt from the effectiveness requirements; it is intended for devices that benefit patients with rare disease.
